# Appraising Neonatal Morbidity and Mortality in a Developing Country Categorized by Gestational Age Grouping and Implications for Targeted Interventions

**DOI:** 10.3389/fped.2022.899645

**Published:** 2022-05-30

**Authors:** Olugbenga Ayodeji Mokuolu, Omotayo Oluwakemi Adesiyun, Olayinka Rasheed Ibrahim, Habibat Dirisu Suberu, Selimat Ibrahim, Surajudeen Oyeleke Bello, Moboni Mokikan, Temitope Olorunshola Obasa, Mohammed Baba Abdulkadir

**Affiliations:** ^1^Neonatal Intensive Care Unit, Department of Pediatrics, University of Ilorin Teaching Hospital, Ilorin, Nigeria; ^2^Department of Pediatrics and Child Health, College of Health Sciences, University of Ilorin, Ilorin, Nigeria; ^3^Centre for Malaria and Other Tropical Diseases Care, University of Ilorin Teaching Hospital, Ilorin, Nigeria; ^4^Department of Biostatistics and Epidemiology, East Tennessee State University, Johnson City, TN, United States

**Keywords:** neonatal mortality, low-middle-income country, preterm, term babies, sustainable development goals

## Abstract

**Introduction:**

Despite the relatively higher neonatal morbidity and mortality in developing countries, there are limited data on the detailed analysis of the burden in Nigeria. With a database of over 14,000 admissions, this study presents a compelling picture of the current trends disaggregated by their gestational age groups. It provides unique opportunities for better-targeted interventions for further reducing newborn mortality in line with SDG 3, Target 3.2.

**Methods:**

This prospective observational study involved newborn babies admitted to the Neonatal Intensive Care Unit of the University of Ilorin Teaching Hospital, Kwara State, Nigeria, between January 2007 and December 2018. The outcome was the neonatal mortality rates. The exposure variables included birth weight, gestational age (preterm versus term), and clinical diagnosis. Frequencies were generated on tables and charts, and the trends or associations were determined.

**Results:**

Of the 14,760 neonates admitted, 9,030 (61.2%) were term babies, 4,847 (32.8%) were preterm babies, and in 792 (5%) of the admissions, the gestational ages could not be determined. Males constituted a higher proportion with 55.9%, and the total number of deaths in the study period was 14.7%. The mortality ratio was highest among babies with a birth weight of less than 1,000 g (38.0%) and gestational age of less than 28 weeks (65.5%). The trend analysis showed that the mortality rate decreased from 17.8 to 13% over the 12 years, *p*-value < 0.0001. For term babies, mortality decreased by 45%, from 15.7% in 2007 to 8.7% in 2018, while the decline in mortality for preterm babies was 28.4%, from 25.7% in 2007 to 18.4% in 2018. For both categories, *p*-values were < 0.001. Regarding morbidity in term babies, asphyxia occurred in (1:3), jaundice (1:5), sepsis (1:6), and respiratory disorders (1:6) of admissions. For mortality, asphyxia occurred in (1:2), sepsis (1:5), jaundice (1:8), and respiratory disorders (1:10) of deaths. The leading causes of morbidity among preterm babies were asphyxia (1:4), sepsis (1:5), respiratory disorders (1:9), and jaundice (1.10). For mortality, their contributions were asphyxia (≈1:2); sepsis (1:5); respiratory disorders (1:9), and jaundice (1:10).

**Conclusion:**

There was a marked improvement in neonatal mortality trends. However, severe perinatal asphyxia, sepsis, hyperbilirubinemia, and respiratory disorders were the leading conditions contributing to 75% of the morbidities and mortalities. Measures to further accelerate the reduction in neonatal morbidity and mortality are discussed.

## Introduction

Globally, substantial progress was made toward the achievement of now rested fourth Millennium Development Goal (MDG) with a reduction of almost half in the number of under-five deaths from 1990 to 2013 (1, 2). During the same period, infant mortality reduced from 63 to 34 per thousand live births, while neonatal mortality rate decreased from 33 to 20 per thousand live births (3). In Nigeria, the neonatal mortality rate also decreased from 52 to 37 per thousand live births (4). To achieve these milestones, the federal government scaled up interventions in 2007 by rolling out the Integrated Maternal, Newborn, and Child Health (IMNCH) strategy, implemented by most states in Nigeria (5–8). The government also introduced the Midwives Service Scheme (MSS) a few years later, focusing on safe delivery, especially in rural areas (9). Furthermore, Nigeria’s partnerships with United States Agency for International Development (USAID), Saving Newborn Lives/Save the Children-US, the Partnership for Reviving Routine Immunization in Northern Nigeria (PRRINN), -WHO, and UNICEF have all contributed to the progress made (5, 10).

Although there is progress in neonatal indices, a more significant burden still occurs in low-and middle-income countries than in high-income countries. In 2017, the neonatal mortality rate was highest in Sub-Saharan Africa (27 deaths per 1,000 live births), about nine times higher than the average of three deaths per 1,000 live births in high-income countries (11). Most neonatal deaths in low-income countries occur in preterm and low birth weight babies. A multi-countries global network study showed the highest mortality rates were for infants born weighing < 1,500 grams, with rates of approximately 700/1,000 births for India and Pakistan. Similarly, the study also showed a higher death rate among those with the lowest gestational age (12).

Appraising neonatal mortality causes in Nigeria, or probably in many developing countries, has been somewhat challenging. Large-scale studies were done using verbal autopsy techniques at community levels. The country’s demographic health survey indicated a slow decline in the neonatal mortality rate from 42 in 1990 to 39 per 1,000 live births in 2018 (4, 13). The studies mentioned above have many limitations with the current data gathering methods. This may explain, for instance, the complete absence of neonatal jaundice and thermoregulatory problems as a cause of mortality in the newborn (13). Furthermore, the contributions of preterm babies and their specific morbidities are often not reflected. Hence a holistic picture of neonatal mortality would require an appraisal of community and hospital-based data stratified by the gestational categories. A few reports have attempted to describe the hospital-based neonatal mortality and morbidity trends. However, they have often included relatively small numbers. They tend to have significant limitations in the disease categorizations, especially among preterm babies (14–18).

The adoption of the Sustainable Development Goals (SDGs) by the United Nations General Assembly appears to be an opportunity to reset and recalibrate several health indices as new targets are rolled out against the target date of 2030. For instance, SDG 3 has as one of its components that;

*“by 2030, end preventable deaths of newborns and children under 5 years of age, with all countries aiming to reduce neonatal mortality to at least as low as 12 per 1,000 live births and under-5 mortality to at least as low as 25 per 1,000 live births”*(19).

The intentions expressed in this goal emphasize the need to end preventable deaths. A hospital-based report showed perinatal rates of 81/1,000 and neonatal mortality rates of 31/1,000 live births (20). Due to differences in access to health and available infrastructures, there are significant variabilities in the causes of morbidity/mortality from place to place. Hence, the need to determine region-specific mortality. We, therefore, hypothesized that the trends of neonatal mortality rate differ over the study period due to the impact of the pragmatic approach. This study presents 12-year prospectively gathered data on neonatal morbidity and mortality, as seen at the University of Ilorin Teaching Hospital, Nigeria. With a database of over 14,000 admissions, with over 4,500 preterm babies, this study presents a compelling picture of a developing country’s current trends among term and preterm babies. It identifies unique opportunities for better-targeted interventions to recalibrate further efforts to reduce newborn mortality in line with SDG 3.

## Methodology

### Study Site

The study was carried out at the neonatal intensive care unit (NICU) of the University of Ilorin Teaching Hospital, Nigeria. Ilorin in Kwara State, Nigeria. The University of Ilorin Teaching Hospital is a 600-bed facility referral hospital. However, due to the weaknesses in the referral systems, it also offers significant degrees of primary and secondary level care services. Hence, a hospital-based study from the facility also significantly reflects the community distribution of newborn morbidity and mortality. The NICU provides level II-a care to newborn babies from the hospital’s labor and delivery unit and outborn babies from other parts of Kwara and the adjoining states (21). The facility has an annual admission rate of 1,300–1,500 newborns (22).

### Study Design

This was a prospective observational study. A special register was created *a priori* to capture all admissions data using the Microsoft Excel software package. The data capture form was printed out and bound for ease of entry by the doctors on the ward. This register was distinct from the regular hospital register of admissions that the nurses often complete. The data capture register was designed to enhance the documentation of important variables like the hierarchy of diagnosis, gestational age, duration of admission, and necessary treatments. All newborn babies admitted at the University of Ilorin Teaching Hospital (UITH) over 12 years (January 2007 to December 2018) had their data entered into this register by unit Junior Residents. The Consultants supervised the data entry and ensured the validity of the information entered. The outcomes were updated at discharge or deaths of the babies.

### Study Participants

The study included all the neonates (inborn and out-born babies) at the UITH during the study period.

### Neonatal Care Practices

Each baby admitted to the Unit received standard care based on its protocols for managing their clinical conditions, including relevant blood culture, complete blood counts, serum bilirubin, and radiological investigations. The gestational ages were assessed using the last normal menstrual period and the new Ballard score within 24 h of admission (23). Unfortunately, the gestational ages could not be documented in some cases, especially when the last menstrual period (LMP) or ultrasound (USS) dates were unavailable. The babies were older than 72 h at presentation. Besides, Lubchenco et al.’s (24) chart was used to classify the babies. Those that presented for admission after 72 h had their gestational age group identified as unclassified. The Lubchenco chart applies to Nigerian newborn babies (25).

Neonatal care is largely anticipatory and mainly addresses the newborn’s basic needs. These needs are;

The need to be pink—This comprises measures taken to ensure adequate cardiopulmonary transition and maintenance through a baby’s stay on the ward. Oxygen delivery is through the appropriate technologies of oxygen concentrators and piped oxygen from the hospital oxygen plant (26). There is limited capacity to support respiration using bubble C-PAP, a BiPAP machine, or IPPV.

The need to be warm—We introduced some innovative measures to create an appropriate mix of modern and relevant technologies to provide a suitable ambiance for the babies; these include digitally recycled incubators and locally fabricated radiant heaters (27). These are used concurrently with standard radiant warmers and modern incubators to provide a neutral thermal environment.

The need to be sweet refers to the provision of adequate calories. Babies with initial respiratory difficulties or other identified contraindications to oral feeds are commenced on glucose infusion after birth. Otherwise, there is an early introduction of trophic feeds using the mother’s breast milk administered as gavage feeding or direct breastfeeding, depending on the babies’ conditions. There was daily monitoring of the babies’ weights until there was consistency in weight gain, and after that, monitored weekly.

The need to be clean—Maintaining asepsis is addressed by observing strict handwashing between patient contacts, restriction of access to the unit, and maintenance of aseptic procedures.

Other care: These are provided according to the specific problems identified in the babies, such as managing hyperbilirubinemia, administration of blood transfusion, or corrective surgical procedures for some congenital or acute surgical conditions.

Upgrade and changes in neonatal care practices: The unit witnessed improved technology and human capacity during the study period. Firstly, the old incubators were refurbished in 2005 with enhanced performance (27). In 2009, the unit also moved to a purposely built ward, and Vamed Engineering Limited supplied and maintained a new set of incubators. In 2011, improvised bulb CPAP was introduced. From 2013 to 2015, the neonatal resuscitation program was held regularly for the NICU and labor ward staff. In addition, a resident was posted to labor to provide support for newborns delivered in the hospital.

Follow-up care: Upon discharge, babies are seen regularly at either of the two clinics. One is the outpatient services operated by the neonatal unit, while the other is the growth and development clinic in the pediatric outpatient department. The length of follow-up is determined by the nature and severity of the admitting condition.

### Definitions of Terms and Criteria Used for Diagnosis

Term babies: Babies born at completed 37 to 42 weeks of gestational age (28).

Preterm babies: Babies born at less than 37 completed weeks (28).

Post-term: Babies born after the completed 37 weeks (28).

Perinatal asphyxia: This was based on the history of failure to cry at birth with evidence of neurological involvement at admission (29).

Sepsis was defined as the presence of signs and symptoms of infections with evidence of systemic inflammatory response on laboratory evaluations (30).

Hyperbilirubinemia: was defined as elevated serum bilirubin based on age and gestational age (31).

Bilirubin encephalopathy: Presence of elevated serum bilirubin with evidence of neurological involvement (31).

### Data Analysis

Admission and hospital data were entered into the specially designed data capture program on a Microsoft Excel spreadsheet in the unit. The data were analyzed weekly at the unit level and presented at the monthly departmental clinical meetings. The data was further analyzed annually. The captured data were exported to SPSS version 20 to generate frequency tables, charts, and the chi-square test for trends.

## Results

### General Characteristics

**FIGURE 1:**
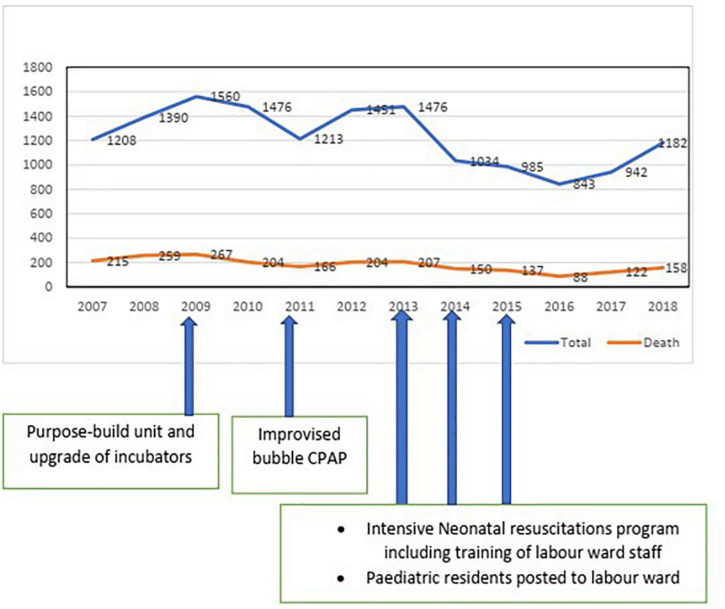
Admission and mortality trends with timing of interventions during the study period.

A total number of fourteen thousand, seven hundred and sixty neonates (newborn babies within the first 28 days of life) were admitted to the NICU during the study period. Males numbered eight thousand, two hundred and fifty-four (55.9%) while females numbered six thousand five hundred and six (44.1%), giving a male to female ratio of 1.3:1. The total number of deaths recorded within the period was two thousand, one hundred and seventy-seven, with a percentage mortality of 14.7%. See Table 1. Overall, there was a significant downward slope in the relative mortality (Figure 1); Chi-square for the trend gave a *p*-value < 0.0001.

**TABLE 1:** Summary of newborn admissions and outcomes (2007–2018).

Parameters	2007	2008	2009	2010	2011	2012	2013	2014	2015	2016	2017	2018	Total
Total admission	1,208	1,390	1,560	1,476	1,213	1,451	1,476	1,034	985	843	942	1,182	14,760
Males	670	811	869	764	693	817	817	603	551	471	527	661	8,254
Females	538	579	691	712	520	634	659	431	434	372	415	521	6,506
Deaths	215	259	267	204	166	204	207	150	137	88	122	158	2,177
%Mortality*	17.8	18.6	17.1	13.8	13.7	13.9	14.0	14.5	14.1	10.4	13.0	13.4	14.7

*Chi-square for linear trend (χ^2^) = 53.0964, df = 1, p = <0.0001.

Of the total admissions of 14,760, there were 9,030 (61.1%) term babies, 4,938 (33.5%) preterm babies, and in 792 (5.4%) of the admissions, the gestational ages could not be determined. These were primarily babies admitted after 72 h with parents not having relevant antenatal information for gestational age assessment. Comparing the contributions of term and preterm babies to admission and mortalities among babies with known gestational ages, the results indicate that term babies accounted for 61.1% of admissions and 49.2% of mortalities, while preterm contributed 32.8% of admissions and 45.2% of mortality (χ^2^ = 179.6; *p* < 0.00001).

### Trends in Mortality Among Term Babies

Term babies (37–42 completed weeks of gestation) were nine thousand and thirty, accounting for 61.1% of the total number of admissions. One thousand and fifty-seven died, giving a case fatality of 11.7% for term babies. There was a downward trend in the annual proportion of mortality among term babies from 15.7% in 2007 to 8.7% in 2018, with chi-square for trend (χ^2^) = (2) = 56.1673 df = 1, *p* < 0.0001. Further sub-analysis of the deaths indicated that for the period 2007–2009, the proportion of deaths for term babies was 12.8% (/2912) while deaths for the period 2013–2018 was 9.4% (412/4409); X^2^ = 555.79, *p* = 0.00001, OR = 1.33 (95% CI: 1.13–1.59)]. However, the mortality rate between 2010–2012 (11.0%; 273/2766) vs 2013–2018 (9.4%; 412/4409) were comparable, 2 = 0.543, *p* = 0.461 (see Table 2).

**TABLE 2:** Admissions and mortality trends in Term babies 2007–2018.

Admission	2007	2008	2009	2010	2011	2012	2013	2014	2015	2016	2017	2018	Total
Term	752	822	966	867	756	870	885	650	604	521	589	748	9,030
Deaths	118	126	128	78	84	111	94	72	62	48	71	65	1,057
% Mortality*	15.7	15.3	13.3	9.0	11.1	12.8	10.6	11.1	10.2	9.2	12.1	8.7	11.7

*Chi-square for linear trend (χ^2^) = 56.1673 df = 1 p < 0.0001.

### Trends in Mortality Among Preterm Admissions

Preterm babies (gestational age less than 37 weeks) were four thousand nine hundred and thirty-eight, representing 33.5% of the total admissions within the period. A total number of nine hundred and ninety-eight preterm babies died over the 12 years of the study, representing 20.2% of their admissions. See Table 3. There was a significant decline in the proportion of preterm mortality relative to their admissions from 25.7% in 2007 to 17.3% in 2017. chi-square for trend (χ2) = 11.1672, df = 1, *p* = 0.0008. Further analysis showed that proportion of death declined from 2007–2009 (20.0%; 347/1737) compared with periods of 2010 to 2012 (15.7%; 234/1255), and 2013 to 2018 (18.2%; 417/2710) with 2 values of 9.861, *p* = 0.002 and 15.670, *p* = 0.001, respectively. However, the proportion of death between 2010–2012 and 2013 to 2018 was comparable (2 = 0.078, *p* = 0.799).

**TABLE 3:** Preterm babies’ admissions and mortality 2007–2018.

Admission	2007	2008	2009	2010	2011	2012	2013	2014	2015	2016	2017	2018	Total
Preterm	452	465	473	481	360	414	459	349	376	322	353	434	4,938
Death	116	116	115	100	63	71	79	70	68	60	60	80	998
% Mortality*	25.7	24.9	24.3	20.8	17.5	17.1	17.2	20.9	18.1	18.6	17.3	20.0	20.2

*Chi-square for linear trend (χ^2^) = 11.1672, df = 1, p = 0.0008.

### Causes of Morbidity and Mortality Among Term Babies

**FIGURE 2:**
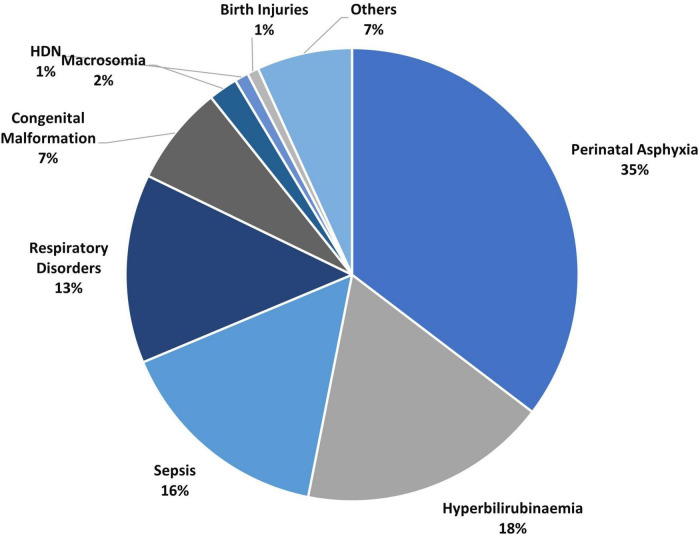
Morbidity pattern among 9,030 term babies.

**FIGURE 3:**
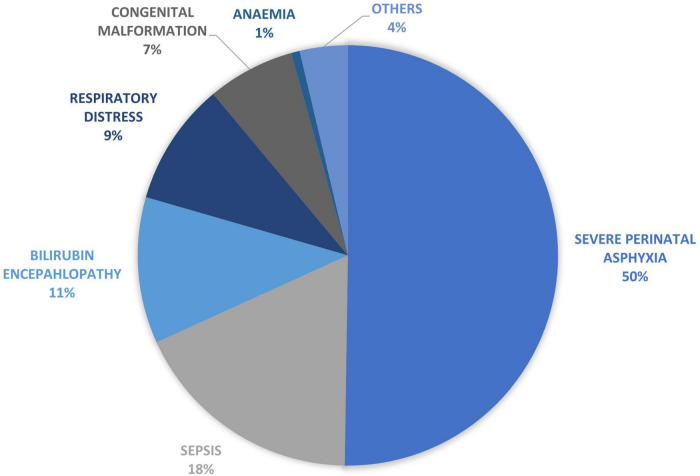
Cause of mortality among term babies.

The leading causes of morbidity among term babies included perinatal asphyxia (PA) (35%), hyperbilirubinemia (18%), neonatal sepsis (16%), respiratory distress (13%), congenital malformation (7%), macrosomia (2%) and others (9%) as shown in Figure 2. The leading causes of death included perinatal asphyxia (50%), neonatal sepsis (18%), bilirubin encephalopathy (11%), respiratory distress (9%), congenital malformation (7%), hematological problems (1%), and others (4%) as shown in Figure 3.

### Causes of Morbidity and Mortality Among Preterm Babies

**FIGURE 4:**
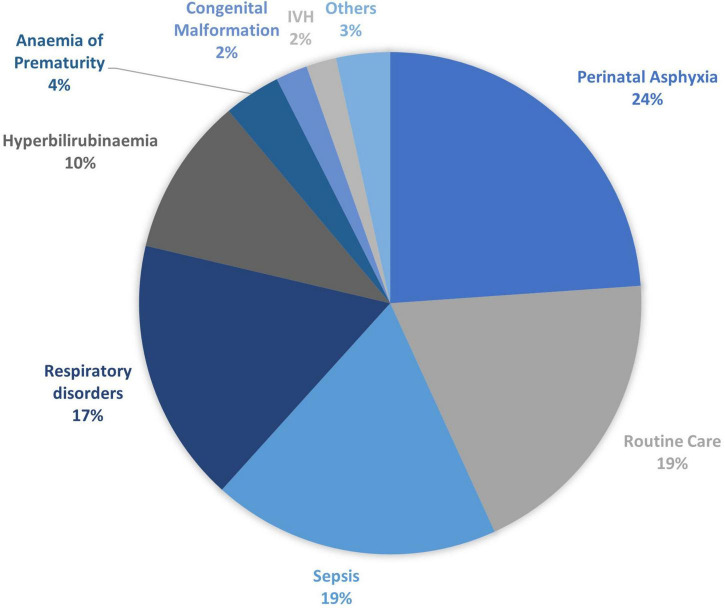
Morbidity pattern among 4,938 preterm babies.

**FIGURE 5:**
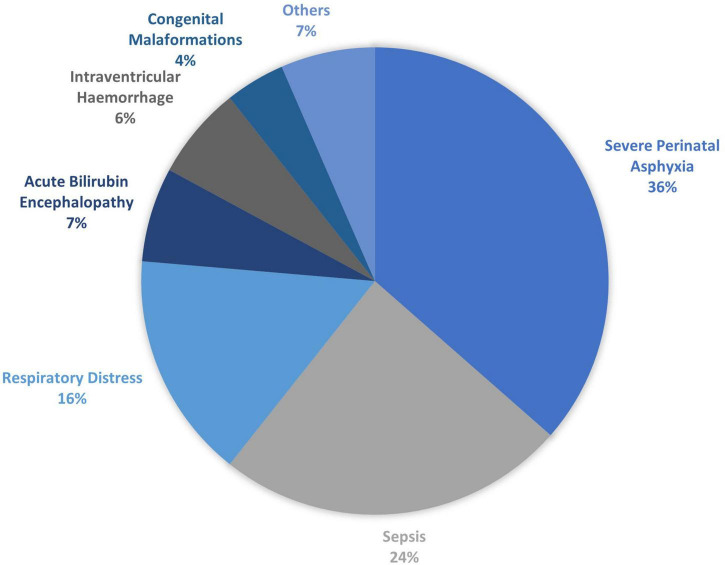
Cause of mortality among preterm babies.

The causes of morbidity among preterm babies included perinatal asphyxia (24%), neonatal sepsis (19%), respiratory distress (17%), hyperbilirubinemia (10%), anemia of prematurity (4%), intraventricular hemorrhage (2%), others (3%) and 19% of the total preterm babies were admitted for routine care (Figure 4). The causes of death included perinatal asphyxia (36%), neonatal sepsis (24%), respiratory distress (16%), bilirubin encephalopathy (7%), intraventricular hemorrhage (6%), congenital malformation (4%) and others (7%) as shown in Figure 5.

### Distribution of Mortality by Birth Weight

**FIGURE 6:**
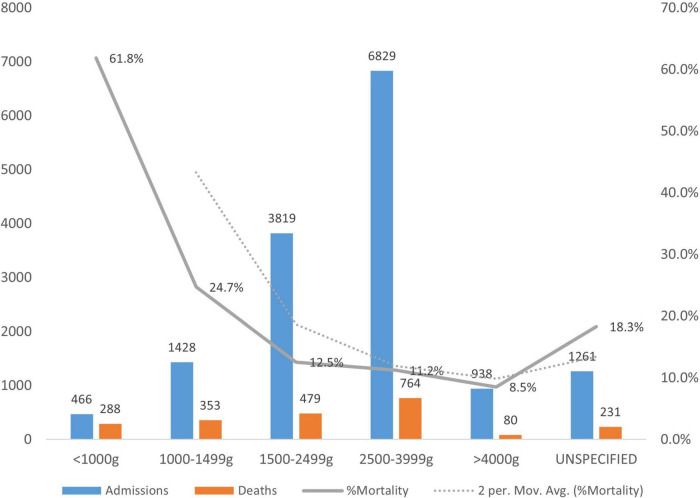
Trend in morbidity and mortality based on birthweight.

The distribution of mortality based on birth weight showed that the highest percentage of mortality (38%) was among newborns who weighed less than 1,000 g, while the lowest rate of mortality (8%) was among babies who weighed ≥ 4,000 g. More details of percentage mortality based on birth weight are shown in Figure 6.

### Distribution of Mortality by Gestational Age

**FIGURE 7:**
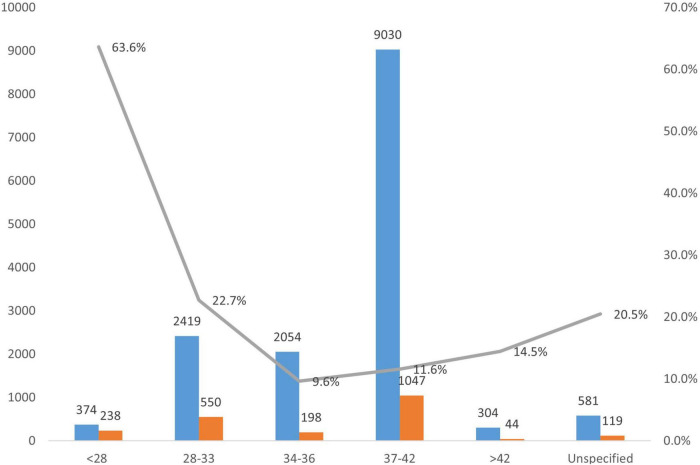
Trend in morbidity and mortality based on gestational age.

The highest percentage of mortality (63.6%) was among babies with gestational age < 28 weeks, while the least percentage of mortality (9.6%) was among those with gestational age 34–36 weeks. The other details are shown in Figure 7.

## Discussion

The present study involved 14,760 newborns over 12 years, and it represents one of the most extensive data sets from tertiary health facilities in a developing country. From the literature search, the closest report regarding the audit of newborn care in a tertiary health center from a developing nation was the retrospective study involving 7,225 newborns over 10 years by Owa and Osinaike (32) in Southwestern Nigeria. This extensive database from a facility that provides considerable access to primary and secondary health care will significantly represent the spectrum of neonatal conditions prevalent in the study site. The precision of diagnosis will also be better than the verbal autopsy techniques, which have been shown to have some challenges, mainly when carried out by non-physicians (31, 32).

Of importance for a study from Nigeria, a middle-income country, causes of morbidity and mortality in this study are reported separately for term and preterm neonates. In prior studies, prematurity itself has often been labeled as the “cause” of morbidity or mortality but our approach sheds additional light on how causes of morbidity and mortality vary between a baby who is term as compared to preterm. The third goal of the SDG is concerned with reducing neonatal mortality to less than 20 per thousand live births, which underscores the need to identify the peculiar characteristics of preterm babies as part of efforts toward meeting this goal. Unfortunately, the WHO annual health statistics and other health indices data continue to lump all causes of newborn deaths together (33). From the robust data in this study, a critical separation of the causes of death into two main categories has allowed the separation of burden and causes of death in preterm babies from term babies. It is expected to serve as a critical reference in tracking the load of preterm babies and the distribution of their various morbidities/mortalities.

The term babies’ admissions trend showed a steady rise from 8.3% in 2007 to a peak at 10.7% in 2009 before a steady decline to 5.7% in 2016. Within the period, the annual percentage mortality compared with the admission of the same year showed a decrease from 15.7% in 2007 to 9.4% in 2018. This finding is also in tandem with Nigeria’s demographic health survey that showed a progressive decline in neonatal mortality in Nigeria (4). However, our results contrast with a similar study in Tanzania, where the mortality rate stagnated over 10 years (34). The significant reduction in mortality among term babies observed between 2013–2018 and 2007–2009 could be attributed to some factors. As part of the movement of the teaching hospital to the current definitive site, the NICU was also relocated at the end of 2009 to a more purpose-built facility that is better aligned with the labor and delivery unit, improved oxygen supply, and acquisition of a modern set of incubators and ventilators through federal government’s VAMED Engineering Equipment Program. The set of modern incubators complemented existing digitally re-cycled old incubators, which have been documented to improve neonatal outcomes significantly (27).

The data on morbidity among term babies revealed that perinatal asphyxia (35%), hyperbilirubinemia (18%), neonatal sepsis (15%), and respiratory distress (14%) were the leading causes of admissions. Congenital malformations, often unreported, were ranked as the 5th most common cause of admissions (2). While virtually all studies that audited neonatal morbidity and mortality have reported perinatal asphyxia as the leading cause of newborn admissions, only a few have reported the burden of neonatal jaundice (14, 15) This has often led to jaundice being a “hidden cause of neonatal morbidity/mortality.” This is not unexpected as most studies did not separate term from preterm babies or were limited by study sample size (18, 35). However, in keeping with the findings of this study are a few studies that examined the actual burden of jaundice among newborns and documented higher prevalence (as high as 25.6 to 45.6%) among newborn admissions (36, 37).

The causes of death among term babies showed that perinatal asphyxia still ranks first, keeping with global causes of newborn deaths in developing countries (2). This was followed by neonatal sepsis, bilirubin encephalopathy, respiratory disorders, congenital malformation, and hematological problems. A systematic review of the burden of neonatal death in the southeast Asian nations showed the leading cause of death as birth asphyxia, neonatal sepsis, and congenital malformation with the absence of hyperbilirubinemia (38). This study shows neonatal jaundice was ranked the third most common cause of death after perinatal asphyxia and neonatal sepsis. Unfortunately, the global efforts focus more on perinatal asphyxia and sepsis, while hyperbilirubinemia remains a cause for concern (39). The pie-chart showing mortality (Figure 2) also demonstrated that congenital malformation was responsible for 7% of deaths, which seems relatively high. Still low compared to figures from Cameroon, which recorded a 10.5% mortality rate (40). These differences could be attributed to the fact that the Cameroon study did not separate preterm from term babies.

From the present study, the trend of annual preterm admissions showed a steady rise with dips in 2011 and 2016. This can be attributed to the hospital’s relocation from the old site to the permanent site and various industrial actions that occurred during the period. Furthermore, the chi-square for trend showed significant improvement in the survival rate among this set of newborns. This can be attributed to the improved newborn care and our ability to adapt where there are challenges (27).

The leading causes of admission among preterm babies (Figure 3) were perinatal asphyxia, neonatal sepsis, respiratory distress, hyperbilirubinemia, anemia of prematurity, intraventricular hemorrhage, and congenital malformation, respectively. This is in contrast to the order of causes of admission (respiratory distress, sepsis, hyperbilirubinemia, and perinatal asphyxia) reported by Kunle-Olowu et al. (40) at a tertiary center in South-South Nigeria. The differences in the order of causes of admission could be partly attributed to the relatively small size of preterm babies (41) studied by Kunle-Olowu et al. (40) compared to the subjects in the present study. Babies admitted for routine prematurity care included those whose gestational age was less than 32 weeks and weighed less than 1,500 g who were offered care to prevent problems associated with prematurity. Unsurprisingly, neonatal sepsis ranked second as the leading cause of admissions which is not unexpected considering their vulnerability to infections. The leading causes of death among the preterm (Figure 3) were perinatal asphyxia, sepsis, respiratory distress, and intraventricular hemorrhage. Though limited in distribution cause of death, a study in Bangladesh identified perinatal asphyxia and sepsis as the leading cause of preterm babies’ death, which is in keeping with our observation (42). This study has shown that perinatal asphyxia is the leading cause of death among newborns, keeping with global findings. In contrast, Ugwu (42) documented respiratory complications as the number one cause of death and perinatal asphyxia as the fourth leading cause of death. The differences in the findings could be because the most common preterm problem may present with respiratory distress, which may lead to classification as a respiratory disorder. While respiratory diseases such as respiratory distress syndrome are common in preterm, various clinical conditions such as aspiration, perinatal asphyxia with pulmonary hypertension, and infection may present with respiratory distress in preterm babies after birth (46).

The birth weight remained a strong determinant of survival of newborns, and low birth weight babies tend to have higher mortality than other weight categories, which is in keeping with a multi-countries study on neonatal death (12). The relatively low survival rate (38%) of extremely low birth weight babies may be attributed to the high rate of complications in this weight category. Furthermore, this study’s finding of least mortality due to macrosomia seems unexpected considering that macrosomic babies are a high-risk group. However, the results could be explained because most macrosomic babies tend to be delivered at a health facility with early admissions. Also supporting the findings was the recent work of Spogmai et al. (43), who found that macrosomia was associated with a generally protective effect against perinatal death.

The survival rate of 36% among babies less than 28 weeks from this study showed that 3–4 of 10 babies survived. Thus, the need for a review of the cut-off gestation age of 28 weeks used to define the period of viability in most developing countries. The least percentage of mortality was also observed at the gestational age of 34–36 weeks which is similar to the findings by Klingenberg et al. (44) at a tertiary health facility in Tanzania. The low mortality among gestational age 34–36 weeks may be related to the fact these babies have achieved lung maturity, a birth weight that favors less difficulty at delivery, and lesser problems associated with prematurity (45). Our findings are consistent with the call by Amadi et al. (26) to use frugal remedies for lowering facility-based neonatal mortality and morbidity in Nigeria. Additionally, Mokuolu et al. (24) provide a rational basis for improved selection and quality of care for pregnant women at risk of preterm delivery.

## Conclusion

The study demonstrated gains in using several pragmatic approaches to the care of newborns despite resource challenges with a decline in the mortality rate over 12 years. In addition, hyperbilirubinemia remains among the leading cause of neonatal morbidity and mortality in our environment. This study also shows that pragmatic approaches remain crucial to the SDG objective of reducing newborn mortality. A unique contribution is reporting specific causes of morbidity and of mortality in two groups—for term babies and also for premature babies. This approach is more informative than simply listing “prematurity” as the cause and will inform future interventions which may differ by gestational age.

### Recommendation

There is the need to build on these gains with the adoption of awareness campaigns to improve neonatal morbidity and mortality at various levels of delivery, greater penetration of essential care of the newborn in the community and lower level of health facilities, timely referrals, and building the skills of frontline pediatricians and neonatologists on ventilatory support, parenteral nutrition, and neonatal resuscitation.

### Limitations

There were years when the hospital experienced prolonged strike actions that disrupted services. While the neonatal unit never stopped operating during those periods, a significant reduction in the number of babies seen and admissions was selectively related to the very severe conditions. This may have caused minor distortions in the denominators used for those years. In addition, 186 babies were delivered outside the hospital and presented late with unknown birth weights. We computed the weight categorization from the weight taken at the point of admission.

## Data Availability Statement

The raw data supporting the conclusions of this article will be made available by the authors, without undue reservation.

## Ethics Statement

The studies involving human participants were reviewed and approved by University of Ilorin Teaching Hospital Ethical Review Committee. Written informed consent from the participants’ legal guardian/next of kin was not required to participate in this study in accordance with the national legislation and the institutional requirements.

## Author Contributions

OM and OA designed the study and developed the initial draft of the manuscript. All authors were part of the data collation and contributed to the review of the final manuscript.

## Conflict of Interest

The authors declare that the research was conducted in the absence of any commercial or financial relationships that could be construed as a potential conflict of interest.

## Publisher’s Note

All claims expressed in this article are solely those of the authors and do not necessarily represent those of their affiliated organizations, or those of the publisher, the editors and the reviewers. Any product that may be evaluated in this article, or claim that may be made by its manufacturer, is not guaranteed or endorsed by the publisher.
